# Is Restless Legs Syndrome De Facto Thyroid Disease?

**DOI:** 10.3390/biomedicines10102502

**Published:** 2022-10-07

**Authors:** Szymon Suwała, Jakub Rzeszuto, Rafał Glonek, Magdalena Krintus, Roman Junik

**Affiliations:** 1 Department of Endocrinology and Diabetology, Nicolaus Copernicus University, Collegium Medicum, 9 Sklodowskiej-Curie Street, 85-094 Bydgoszcz, Poland; 2 Evidence-Based Medicine Students Scientific Club of Department of Endocrinology and Diabetology, Nicolaus Copernicus University, Collegium Medicum, 9 Sklodowskiej-Curie Street, 85-094 Bydgoszcz, Poland; 3 Department of Laboratory Medicine, Nicolaus Copernicus University, Collegium Medicum, 9 Sklodowskiej-Curie Street, 85-094 Bydgoszcz, Poland

**Keywords:** restless legs syndrome, thyroid, autoimmunology

## Abstract

While a primary role in the pathogenesis of restless legs syndrome (RLS) has been attributed to dysfunction of the dopaminergic system and impaired iron metabolism (particularly in the central nervous system), it has been hypothesized that an imbalance between thyroid hormones and dopaminergic activity may be the starting point for all aspects of RLS. Although this hypothesis was proposed more than a decade ago, it has not yet been verified beyond doubt. The main aim of this study is to compare the prevalence of RLS in a population of patients with the most common thyroid gland diseases with a population of individuals with a healthy thyroid gland. The study included 237 participants divided into smaller groups according to the thyroid disease concerning them. Each participant had a laboratory diagnosis, an ultrasound scan and an assessment of the fulfilment of RLS criteria according to the International Restless Legs Syndrome Study Group (IRLSSG) criteria. The results obtained were subjected to statistical analysis. RLS is significantly more common in patients with known thyroid disease; Hashimoto’s disease, among others, manifests a 2.56× higher risk of a positive diagnosis for RLS than the general population. The association of RLS with thyroid disease is notable, although it is difficult to conclude unequivocally that there is a cause-and-effect relationship between the two. Further investigation into a potentially autoimmune cause of restless legs syndrome should be considered.

## 1. Introduction

Restless legs syndrome (RLS) is a musculoskeletal disorder characterized by an unpleasant sensation of the urge to move the lower limbs, occurring mainly in the evening and at night, at rest and disappearing with movement. It affects 7.2–24.1% of the population in Western countries [1,2] (in contrast to Asia, where the prevalence is estimated at 0.1–12% [3,4,5,6,7]), and is more common in women (2:1 in relation to men, probably due to a link between dopaminergic and estrogenic effects [8,9]), pregnant women, the elderly and patients with iron deficiency anemia or renal failure [10]. According to current knowledge, a primary role in the pathogenesis of RLS is attributed to dysfunction of the dopaminergic system, impaired iron metabolism (especially in the central nervous system) and, not least, genetic susceptibility (RLS is the first such common sleep disorder for which genome-wide association studies have been conducted and genetic risk loci identified [11]).

However, in 2010, Pereira et al. announced the hypothesis that the starting point for all aspects of restless legs syndrome may be an imbalance between thyroid gland hormones and dopaminergic system activity [12,13]. One of the basic assumptions of this hypothesis was that in the course of RLS, dopamine is not secreted in a sufficient quantity that would be able to inhibit TSH release from pituitary thyrotropic cells. Thyroid hormones plasma level/release, through a feedback mechanism, would be then increased, and would reduce the perceptual threshold for somatosensory stimuli in the lower limbs, accelerating the flow of signals through synapses on their way to the somatosensory cortex, producing an unpleasant sensations typical for RLS [14]. In further consideration of this hypothesis, a far-reaching assumption was made: restless legs syndrome may be a very mild form of thyrotoxicosis with a specific evening and nighttime increase in sensorimotor complaints [13]. Interestingly, this hypothesis has not been clinically verified to date.

The main aim of the present study is to compare the prevalence of RLS in a population of patients with the most common thyroid gland diseases to a population of individuals with a healthy thyroid, and thus to try to clinically validate the Pereira hypothesis.

## 2. Materials and Methods

In the initial stage of recruiting participants for the study, 390 patients (mainly from the clinic’s endocrinology outpatient clinic) aged 18 to 82 years were contacted. Patients were familiarized with the rules of the study, and 63 patients dropped out of the further qualification stages. The 327 patients remaining in stage II were reviewed for exclusion criteria: currently ongoing pregnancy, a history of or currently active one of the diseases requiring clinical differentiation from RLS (e.g., iron deficiency anaemia, renal failure, diabetes mellitus, Parkinson’s disease, venous or arterial circulatory insufficiency, rheumatoid arthritis, osteoarthritis, fibromyalgia, epilepsy, multiple sclerosis, ADHD, anxiety disorders, obstructive sleep apnea, paraneoplastic syndrome, eating disorders, infections, generalized atherosclerosis) and/or the use of medications and substances that may induce or exacerbate symptoms of RLS (e.g., antihistamines, dopamine antagonists, serotonin reuptake inhibitors, tricyclic antidepressants, lithium, caffeine). Each of the invited study participants, who were not excluded on the basis of their clinical history, had biological material collected for tests that could exclude a possible secondary course of RLS-related symptoms (including peripheral blood morphology, glucose, creatinine with estimated glomerular filtration rate eGFR, ALT, AST, iron, ferritin, ionogram, serum magnesium and total serum calcium) and to assess thyroid function and immunology (TSH, fT_3_, fT_4_, antibodies to thyroid peroxidase, thyroglobulin and the receptor for TSH). An exclusion criterion in patients with thyroid disease was also an iatrogenic cause of the condition, thus including a condition after thyroidectomy, 131I treatment, etc. In addition, patients with already diagnosed restless legs syndrome could not participate in the study, as the aim was to verify whether RLS symptoms appeared after the diagnosis of thyroid disease.

Ultimately, 244 patients were enrolled in the study, and the final analysis considered the results of 237 patients (two patients dropped out, five patients died during the study due to SARS-CoV-2 virus infection, COVID19).

**Figure 1:**
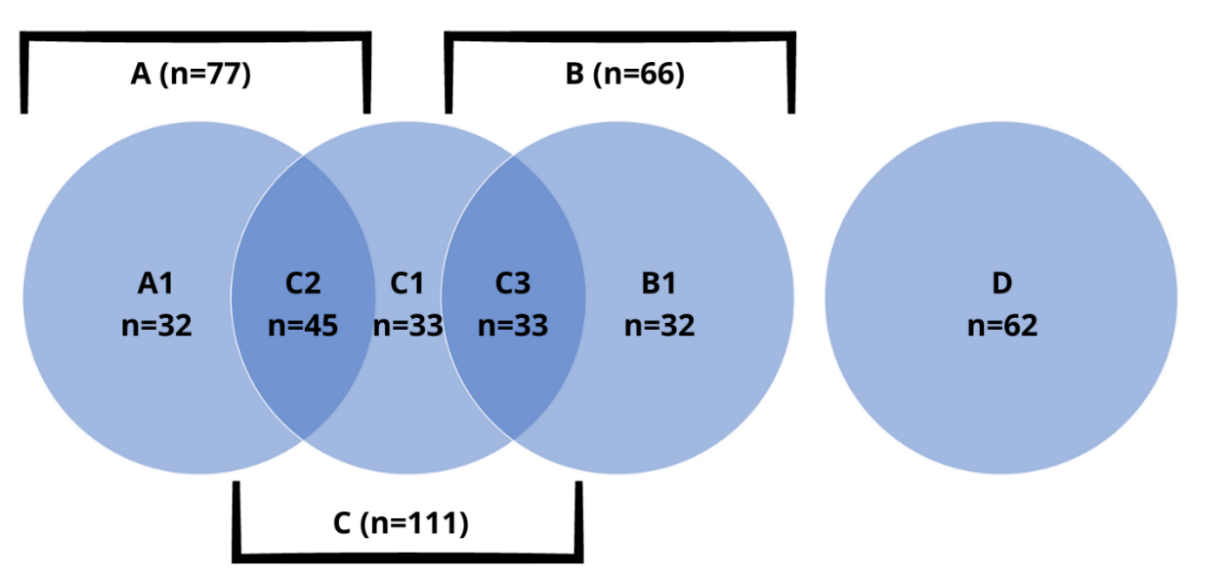
Groups and subgroups of patients, with exact number of subjects.

Eligible patients were divided into the following groups: patients with hypothyroidism (A), hyperthyroidism (B), autoimmune thyroid disease (AITD) (C), and a control group (D, consisting of patients with negative diagnostic observations for endocrine diseases). However, in view of the fact that autoimmune thyroid diseases all too often co-occur with thyroid disorders, it was decided to divide the aforementioned into smaller subgroups: A1 (hypothyroidism without features of AITD), B1 (hyperthyroidism without features of AITD), C1 (AITD without other thyroid disorders), C2 (hypothyroidism in the course of AITD, i.e., Hashimoto’s thyroiditis), C3 (hyperthyroidism in the course of AITD, i.e., Graves’ disease) and D (control group). Exact number of subjects is shown in Figure 1.

Each patient underwent ultrasound examination of the thyroid gland, and weight and height were measured to assess BMI. Each patient was then verified for a diagnosis of RLS, according to current International Restless Legs Syndrome Study Group (IRLSSG) criteria: (1) an urge to move the legs usually but not always accompanied by or felt to be caused by uncomfortable and unpleasant sensations in the legs; (2) the urge to move the legs and any accompanying unpleasant sensations begin or worsen during periods of rest or inactivity such as lying down or sitting; (3) the urge to move the legs and any accompanying unpleasant sensations that are partially or totally relieved by movement, such as walking or stretching, at least as long as the activity continue; (4) the urge to move the legs and any accompanying unpleasant sensations during rest or inactivity that only occur or are worse in the evening or night than during the day; (5) the occurrences of the above features is not solely accounted for as symptoms primary to another medical or behavioral condition (e.g., myalgia, venous stasis, leg edema, arthritis, leg cramps, positional discomfort, habitual foot tapping) [15]. Each of the patients who met the outlined criteria was referred for a consultation by a neurologist to confirm the diagnosis (and start appropriate treatment after project, naturally).

The results obtained were subjected to statistical analysis using Microsoft Excel 2019 (Microsoft Corp, Redmond, WA, USA) and the STATISTICA 13.0 PL statistical package. A significance level of α = 0.05 was adopted for all statistical analysis.

## 3. Results

### 3.1. Characteristics of the Study Group

The entire study group consisted of 237 patients, mostly women (73%; n = 173), with an average age of 38.3 ± 12.1 years and a BMI of 27.2 ± 5.8 kg/m^2^. Each of the subgroups studied differed significantly in the percentage of men and women (in subgroup A1 *p* = 0.019; in B1 *p* = 0.023, C1, C2, C3 and D *p* < 0.001), while there were no significant differences between the subgroups in terms of average age or BMI. A detailed characterization of the subgroups in this respect is presented in Table 1.

When looking for gender differences in laboratory results across the group, these were found in average hemoglobin (13.2 ± 1.0 g/dL in women vs. 13.8 ± 0.9 g/dL in men; *p* = 0.027) and ferritin (48.5 ± 41.4 μg/dL in women vs. 77.0 ± 62.0 μg/dL in men; *p* = 0.013) concentrations. Age correlated positively with chloremia (R = 0.27; *p* = 0.021) and negatively with ferritin concentration (R = −0.24; *p* = 0.044), while BMI correlated negatively with fT_3_ concentration (R = −0.39; *p* < 0.001) and estimated glomerular filtration rate (R = −0.37; *p* = 0.003). No other statistically significant differences were identified.

The different patient subgroups were not found to differ significantly in most of the laboratory parameters determined. The exceptions were some of the peripheral blood count parameters (red blood count: RBC, hematocrit, platelets: PLT, red blood cell distribution width—coefficient of variation: RDW-CV), natriemia, TSH and fT_3_ level, although these were only found in the subgroups belonging to the broader group C. These differences are shown in Table 2.

In the ultrasound examinations of the thyroid gland performed on the patients, the average thyroid volume was 14.7 ± 7.0 mL—a hypoechoic parenchymal picture predominated (75.9%).

Table 3 shows the average thyroid volumes per subgroup; significant statistical differences were found between subgroups A1 and C1 (*p* = 0.038), B1 and C2 (*p* < 0.001), B1 and C3 (*p* = 0.021), C1 and C2 (*p* < 0.001), C2 and C3 (*p* < 0.001) and C2 and D (*p* < 0.001). Thyroid volume negatively correlated with TSH concentration (R = −0.352; *p* = 0.004), but only in general. When it was attempted to assess this correlation in individual groups, it turned out to be statistically insignificant. 21.94% of the respondents were characterized by the presence of focal changes—the studied subgroups did not differ in this percentage in a statistically significant manner.

### 3.2. Characteristics and Epidemiology of Restless Legs Syndrome

All criteria for the IRLSGG diagnosis of RLS were met by 17.3% of subjects (n = 41), including 18.9% of all patients with thyroid disease (n = 33) and 12.9% of controls (n = 8). However, the difference between these groups was not statistically significant (*p* = 0.029). However, when comparing the prevalence of RLS across subgroups, significant differences were identified (*p* = 0.007). Patients in subgroup C2 were most commonly affected by RLS (28.9%; n = 13), followed equally by patients in subgroups C1 and C3 (18.2%; n = 6), 15.6% in subgroup A1 (n = 5), 12.5% in subgroup B1 (n = 4) and finally 11.3% of group D, i.e., the control group (n = 7).

**Figure 2:**
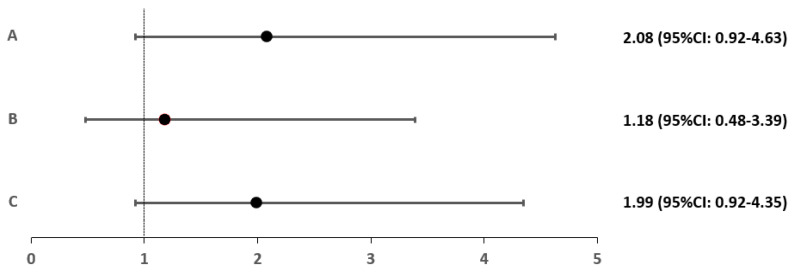
Relative risk of RLS occurrence in groups A, B and C.

**Figure 3:**
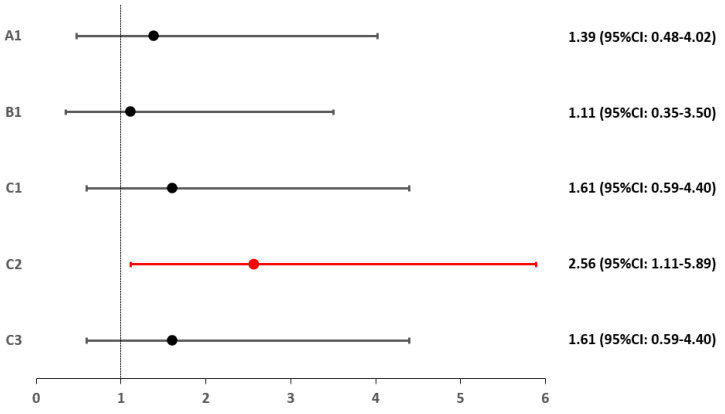
Relative risk of RLS occurrence in subgroups A1, B1, C1, C2 and C3.

For each group and subgroup, a relative risk (RR) index was calculated relative to control group D. Only patients with Hashimoto’s hypothyroidism had a significantly higher risk of RLS (2.56-fold) (*p* = 0.027). This is detailed in Figure 2 and Figure 3.

Overall, subjects diagnosed with RLS were characterized by predominantly female gender (20.3% vs. 8.3%; *p* = 0.033), significantly higher mean age (41.9 ± 10.9 vs. 37.1 ± 12.6; *p* = 0.028), lower MCV (86.9 ± 3.9 vs. 88.5 ± 3.4 fl; *p* = 0.027), lower natremia (141.7 ± 1.1 vs. 142.3 ± 1.4 mmol/L; *p* = 0.022) and lower ferritin levels (36.4 ± 35.3 vs. 59.3 ± 50.2 μg/dL; *p* = 0.026). Focal thyroid lesions also co-occurred more frequently in patients with RLS (38.5% vs. 11.1%; *p* = 0.026).

Analyzing each group and thyroid subgroup separately, further differences were identified between those with and without RLS. These related to the age of the patients, the volume of their thyroid gland and the following determinations: peripheral blood count, sodium, total calcium, magnesium, creatinine, ferritin, ALT, AST, TSH and fT_3_. This is shown in detail in Table 4 and Table 5.

As Table 4 and Table 5 show, smaller thyroid volume was a differentiating feature of patients in group A (with hypothyroidism, regardless of autoimmune etiology) and D (control) and subgroup C2 (with autoimmune thyroid disease, without endocrine disruption). Analysis of thyroid parenchyma echogenicity, flow and the presence of focal lesions in each group showed no significant differences in any of the groups or subgroups.

## 4. Discussion

The present study focused on trying to prove whether there is an association between RLS and thyroid gland diseases. The IRLSSG criteria for the diagnosis of restless legs syndrome were met by 18.9% of all patients with thyroid disease and 12.9% of controls; the most common coincidence being hypothyroidism in the course of Hashimoto’s disease.

Interestingly, we did not find many works on the association between RLS and thyroid disease. Researchers from Singapore attempted to compare epidemiology of RLS between patients with biochemically proven thyroid dysfunctions and control group, however, none of the patients with hypothyroidism or hyperthyroidism met all necessary criteria for the diagnosis of RLS from 1995. When each of the criteria was compared separately, it turned out that 8.2% of patients with thyroid disease met most of the criteria, compared to just 0.9% of the control group of this study, and the patients were not diagnosed with any particular thyroid diseases [16]. In Nada Ahmed’s 2021 study, patients with hypothyroidism were more likely to show features of RLS compared to the general population (14.3% vs. 8.1%), and an autoimmune background of hypothyreosis was also more frequently found in patients with RLS (29.7% vs. 11.0%) [17]. Geng et al. found that patients with RLS were up to eight times more likely to develop thyroid dysfunction (especially hypothyroidism) compared to the control group [18]. Researchers from Sao Paulo, on the other hand, assessed the prevalence of RLS among patients with hyperthyroidism in the course of Graves’ disease. They found that 11.1% of patients with this disease exhibited RLS symptoms secondary to it [19]. In the context of the association between RLS and thyroid disease, the question of the autoimmune basis of thyroid disease is quite often raised. It is therefore worth considering whether perhaps it is the role of the immune system that is central to the mechanism of RLS development, especially as this idea is by no means new, and some researchers have already attempted to verify this question [20]. Is it possible, then, that RLS is just another of the many autoimmune diseases we already know about?

One example of an autoimmune disease is systemic lupus erythematosus (SLE). A 2015 Turkish study showed that RLS is more common in patients with SLE than in the general population (30.6% vs. 4.3%), especially when accompanied by iron deficiency anemia [21]. Similar results were achieved in a 2018 U.S. study (34% vs. 10%) and a 2021 Romanian study (34.2% vs. 7.69%) [22,23]. It is speculated that in genetically susceptible patients, antibodies against cells responsible for dopaminergic signaling in the central nervous system may play a predominant role in the coincidence of RLS with SLE [24]. In 2009, Canadian researchers in a group of 148 patients with rheumatoid arthritis (RA) and 45 with osteoarthritis confirmed that patients with RA were more likely to experience RLS (27.7% vs. 24.4%). The reason for this correlation remains unclear—the influence of subclinical iron deficiency in dopaminergic neurons (common to both disease entities) is most often mentioned in the discussion, but further research is needed, supplemented by analysis of other factors, such as the impact of a malfunctioning immune system [25]. As an autoimmune disease, ankylosing spondylitis is also associated with a higher prevalence of restless legs syndrome than in the healthy population (30.8% vs. 13.2%) [26,27]. Another such disease is psoriasis and psoriatic arthritis (17–68% vs. 4–20%) [28,29]. In this case, researchers have not been able to come to a clear conclusion as to the cause of this correlation, and it has been suspected, as with other diseases, that the problem lies in impaired iron metabolism, but this has not been definitively confirmed.

In celiac disease, RLS has been found to occur more frequently in patients (especially when accompanied by iron deficiency anemia), and the use of a gluten-free diet after 6 months results in a reduction of symptoms in about half of patients [30,31,32].

RLS also co-occurs more frequently in the course of multiple sclerosis (22–22.8% vs. 10% in control group) [33,34]. Research by Vávrova et al. has shown that related gene polymorphisms, such as MAP2K5/SCOR1, may be a potential reason for this, and an association with HLA B51 sometimes linked to many autoimmune diseases (most notably Behçet’s disease) is also suspected [35].

Worthy of note is the increased prevalence of both RLS and thyroid diseases in females [9]. From among many reasons given for this correlation, the effect of estrogens seems to play the major role. For example, in pregnancy while estrogens plasma levels elevates, the incidence of RLS and thyroid disease (especially hypothyroidism) increases. Estrogens increase the concentration of thyroxine binding globulin (TBG) by increasing the content of sialic acid in the molecule, which leads to an increase of TBG-related hormones plasma levels. To maintain an appropriate plasma level of thyroid gland hormones, their secretion must be exaggerated and may be sometimes compromised. It is worth noting that Kulkarni et al. proved that estrogens may act as dopamine antagonists in schizophrenia [36]. Moreover, it is hypothesized that estrogens act similarly in RLS [8]. The association with pregnancy is attributed not only to estrogens plasma levels, but also simultaneously to decreasing iron plasma level during gestation [9], which seems to be another nodal point of both diseases: RLS and thyroid gland disfunctions. The role of iron metabolism in RLS has already been mentioned, but it is necessary to emphasize that iron deficiency may contribute to compromised synthesis of thyroid hormones, their storage and their secretion, due to either decreased oxygen transport or dysfunction of heme-dependent thyroid peroxidase [37]. In conclusion, it cannot be stated that the increased prevalence of RLS in females is uniquely related to thyroid diseases, but undoubtedly, an intersection of their etiopathogenesis occurs.

The selection of laboratory parameters assessed in our subjects was not random. It oscillated, on the one hand, around commonly available tests and, on the other hand, took into account those important for the diagnosis, monitoring, exclusion and differentiation of restless legs syndrome and included full thyroid function tests. The catalogue of tests could have been much broader, but assuming the need to examine a certain number of people and the cost of the tests, it was decided to select them carefully.

Restless legs syndrome all too often co-exists with iron deficiency anemia (considered a secondary cause of RLS), characterized by reduced MCV (mean corpuscular volume) and ferritin, in addition to the obvious decrease in hemoglobin and iron [38,39,40,41,42]. Rabindrakumar et al. showed that, with 70% sensitivity and 40% specificity, certain parameters of a certain value, including MCV and MCHC, can be prognostic factors and precede early iron deficiency anemia—namely: Hb < 12.2 g/dL, MCV < 83.2 fl, MCH < 26.9 pg and MCHC < 33.2 g/dL [43]. In our study, we did not find that any group of patients with RLS (whether general or more specific) was characterized by reduced strictly iron levels. The situation was quite different with MCV (lower in the general sample of patients with restless legs syndrome, as well as in the groups of patients with hyperthyroidism and in the control group), MCHC (reduced in the control group) and ferritin (significantly reduced in the group of patients with autoimmune thyroid disease, especially in the course of Hashimoto’s disease, but also in hypothyroidism without an apparent autoimmune background). Perhaps these patients will develop overt iron deficiency anemia in the future, and at the time of the study showed laboratory, subclinical features of iron deficiency anemia while at the same time the negative effect on the iron-dependent dopaminergic system had already begun. In doing so, it is worth highlighting the fact that microcytic anemias (of which iron deficiency anemia is one) are the most common co-morbidities with thyroid disease [44,45]; another nodal point to bear in mind when analyzing the association of thyroid disease with RLS. Platelet parameters such as, for example, mean platelet volume MPV (elevated in the AITD group without features of endocrine disruption) may have an important function in the pathomechanism of RLS based on increased platelet turnover, coagulation tendency, increased sympathetic activity and oxidative stress, as confirmed by subsequent studies [46,47,48,49].

Patients with restless legs syndrome in the present study were characterized by reduced natremia—a situation not previously reported in the literature. This is intriguing, as the reverse relationship would be expected. After all, it is dopamine and its agonists used in the treatment of RLS that can potentiate the secretion of antidiuretic hormone (acting via aminobutyric acid and dopamine receptors in the suprachiasmatic nucleus), thereby causing the syndrome of inappropriate antidiuretic hormone secretion causing hyponatremia [50].

Another interesting observation from the present study, is the differentiation of the RLS(+) and RLS(-) groups in terms of liver aminotransferase concentrations—higher ALT (although still within normal limits) characterized patients with hypothyroidism in general (group A), but also Hashimoto’s disease (C2) and autoimmune thyroid disease without endocrine disruption (C1). As a general rule, we will not find many similar results in the studies compiled to date [49,51,52], although, on the other hand, it is worth noting that chronic liver disease is among those associated with a higher prevalence of RLS features than in the general population. So, too, does renal failure, especially at the stage of extreme renal failure with high creatinine levels, requiring the implementation of hemodialysis [53,54,55,56]. Interestingly, however, Song Mao’s meta-analysis of twenty-three studies looking for risk factors for RLS in hemodialysis patients did not confirm that creatinine concentration was to be such a risk factor, unlike, for example, hemoglobin and iron concentrations [57]. This raises the suspicion that in renal failure, the crux of the problem of RLS pathogenesis is actually not the fact of impaired filtration per se, but the iron status of the body accompanying this impaired filtration [54].

Strictly thyroid parameters that differentiate patients with restless legs syndrome between groups are fT_3_ concentrations (lower in the group associated with non-autoimmune hyperthyroidism), thyroid volume (lower among patients with Hashimoto’s disease) and the presence of focal lesions in the overall study sample (three times higher in RLS patients than in healthy controls). Few papers related to restless legs syndrome have assessed potential differences in peripheral thyroid hormone levels, but one Turkish case did so, finding a borderline significant difference (lower fT_3_ levels in RLS patients: 4.32 ± 0.60 vs. 4.62 ± 0.67 pmol/L; *p* = 0.067) [49]. Many papers, in turn, emphasize the great importance of the active form of T3 in the generation and maturation of neurons, myelination of axons and ensuring an adequate rate of information flow through nerve cells, if only through the reorganization of extracellular matrix proteins and proteoglycans, an essential substrate for neuronal network differentiation [58,59,60]. Elevated fT_3_ concentrations may also act antagonistically to neuronal development and plasticity by affecting the expression of hypoxia-related genes, but also in relation to the often concomitant iron deficiency [61]. The thyroid hypotrophy found in patients with restless legs syndrome, naturally associated with an active autoimmune process of the thyroid gland, suggests what was mentioned earlier—the potential influence of immune system damage in the role of RLS. This issue definitely requires further, targeted research. What is noteworthy is that the above observation regarding differentiation in relation to thyroid volume and nodular thyroid disease seems to be a new one, previously unreported in the literature.

## 5. Conclusions

RLS is more common in patients with known thyroid disease—most commonly in patients with hypothyroidism in the course of Hashimoto’s disease. Patients with RLS are predominantly characterized by female gender, older average age, lower average red blood cell volume (MCV), lower natremia, higher calcemia, lower ferritin levels, lower thyroid gland volume and a higher incidence of nodular thyroid disease. On the basis of the present study, it is not possible to make a definitive statement on the validity of the hypothesis by Pereira et al. regarding a potentially thyroid-related etiopathogenesis of RLS. However, in conjunction with existing knowledge of RLS, it is possible to hypothesise that restless legs syndrome may have an autoimmune basis, which requires further extensive and in-depth scientific analysis.

## Figures and Tables

**Table 1:** Characteristics of the studied subgroups in terms of sex, age and BMI.

Subgroup (N)	Sex (Female vs. Male)	Average Age in Years (±SD)	Average BMI in kg/m^2^ (±SD)
A1 (32)	81.3% (26) vs. 18.7% (6)	40.5 ± 10.2	29.9 ± 7.1
B1 (45)	71.1% (32) vs. 28.9% (13)	38.6 ± 12.8	27.6 ± 3.4
C1 (33)	87.9% (29) vs. 12.1% (4)	39.3 ± 13.1	25.7 ± 5.5
C2 (33)	93.9% (31) vs. 6.1% (2)	39.5 ± 12.3	25.7 ± 5.7
C3 (32)	90.6% (29) vs. 9.4% (3)	36.6 ± 14.4	28.3 ± 7.4
D (62)	41.9% (26) vs. 58.1% (36)	35.2 ± 14.0	25.8 ± 5.7

**Table 2:** Differences between the C1, C2 and C3 subgroups in selected peripheral blood and biochemical parameters (ns—not significant).

Parameter	C1 vs. C2 (*p* Value)	C1 vs. C3 (*p* Value)	C2 vs. C3 (*p* Value)
RBC [×10^6^/μL]	ns (*p* = 0.991)	4.5 ± 0.3 vs. 4.3 ± 0.3 (*p* = 0.016)	4.5 ± 0.5 vs. 4.3 ± 0.3 (*p* = 0.049)
Hematocrit [%]	ns (*p* = 0.676)	39.8 ± 3.0 vs. 38.1 ± 2.5 (*p* = 0.048)	40.1 ± 3.3 vs. 38.1 ± 2.5 (*p* = 0.030)
PLT [x 10^3^/μL]	ns (*p* = 0.276)	274.3 ± 63.34 vs. 236.2 ± 52.5 (*p* = 0.042)	ns (*p* = 0.278)
RDW-CV [%]	ns (*p* = 0.836)	12.8 ± 0.7 vs. 12.3 ± 0.7 (*p* = 0.040)	12.9 ± 0.7 vs. 12.3 ± 0.7 (*p* = 0.015)
serum sodium [mmol/L]	ns (*p* = 0.444)	143.1 ± 1.1 vs. 141.6 ± 0.7 (*p* = 0.028)	ns (*p* = 0.269)
TSH [uIU/mL]	1.5 ± 0.7 vs. 2.1 ± 1.2 (*p* = 0.018)	ns (*p* = 0.729)	ns (*p* = 0.090)

**Table 3:** Average volumes of the thyroid gland in the studied subgroups.

Subgroup (N)	Average Thyroid Volume in cm^3^ (±SD)
A1	12.0 ± 2.1
B1	14.3 ± 6.0
C1	16.4 ± 4.5
C2	9.3 ± 4.8
C3	18.9 ± 9.8
D	17.6 ± 6.5

**Table 4:** Statistically significant differences between patients with and without RLS, distinguished into groups A, B and C.

Parameter	Patients with RLS	Patients without RLS	*p* Value
Group A			
thyroid volume [cm^3^]	6.1 ± 4.1	10.8 ± 4.1	0.008
MCHC [g/dL]	34.4 ± 0.3	33.8 ± 0.8	0.035
ALT [U/L]	18.9 ± 8.1	13.9 ± 5.4	0.041
Group B			
creatinine [mg/dL]	0.9 ± 0.1	0.7 ± 0.2	0.002
fT_3_ [pg/mL]	2.8 ± 0.2	3.3 ± 0.3	0.004
Group C			
ALT [U/L]	18.2 ± 8.8	14.4 ± 4.3	0.011
AST [U/L]	25.1 ± 16.9	19.5 ± 5.0	0.021
calcium [mmol/L]	2.3 ± 0.1	2.2 ± 0.1	0.003
ferritin [μg/dL]	35.6 ± 26.7	62.2 ± 49.6	0.022
fT3 [pg/mL]	2.9 ± 0.4	3.1 ± 0.3	0.009
Group D			
thyroid volume [cm^3^]	13.18 ± 4.44	18.73 ± 6.37	0.026
MCV [fl]	85.00 ± 3.37	88.38 ± 2.83	0.006
MCHC [pg]	28.48 ± 1.48	29.81 ± 0.97	0.004
PDW [fl]	14.25 ± 1.36	12.53 ± 2.10	0.036
magnesium [mmol/L]	0.69 ± 0.02	0.76 ± 0.04	0.002

**Table 5:** Statistically significant differences between patients with and without RLS, distinguished into subgroups A1, B1, C1, C2 and C3.

Parameter	Patients with RLS	Patients without RLS	*p* Value
Subgroup A1			
ferritin [μg/dL]	35.4 ± 28.4	60.9 ± 50.8	0.026
Subgroup B1			
MCV [fl]	86.5 ± 3.4	88.9 ± 3.9	0.023
fT_3_ [pg/mL]	2.7 ± 0.3	3.2 ± 0.2	0.004
Subgroup C1			
PDW [fl]	11.9 ± 1.2	13.3 ± 1.7	0.045
MPV [fl]	11.0 ± 0.8	10.2 ± 0.2	0.021
P-LCR [%]	27.1 ± 3.9	33.3 ± 6.3	0.019
calcium [mmol/L]	2.3 ± 0.1	2.2 ± 0.1	0.001
Subgroup C2			
thyroid volume [cm^3^]	6.1 ± 4.1	10.5 ± 4.5	0.022
MCHC [g/dL]	34.4 ± 0.3	33.8 ± 0.8	0.030
Subgroup C3			
creatinine [mg/dL]	0.8 ± 0.1	0.6 ± 0.2	0.002

## Data Availability

The data can be made available upon reasonable request—please contact correspondence author.
